# Partial purification and characterization of phytase from *Aspergillus foetidus* MTCC 11682

**DOI:** 10.1186/s13568-018-0725-x

**Published:** 2019-01-04

**Authors:** Sreeja Ajith, Jyotirmoy Ghosh, Divya Shet, S. ShreeVidhya, B. D. Punith, A. V. Elangovan

**Affiliations:** 1 ICAR-National Institute of Animal Nutrition and Physiology, Bangalore, India; 2 Department of Microbiology, Jain University, Bangalore, India; 3 Department of Biotechnology, Jain University, Bangalore, India

**Keywords:** Phytase, *Aspergillus foetidus*, Phy gene

## Abstract

Phytase is a phosphatase enzyme widely used as feed additive to release inorganic phosphorus from plant phytate and enhance its uptake in monogastric animals. Although engineered fungal phytases are used most, a natural enzyme gives opportunity to understand novel properties, if any. In the current study, a novel fungal strain, *Aspergillus foetidus* MTCC 11682 was immobilized on poly urethane cubes and used for phytase production, purification and molecular characterization. Phytase produced by this method was partially purified by ammonium sulphate precipitation and Sephacryl S-200HR gel filtration to 23.4-fold (compared to crude extract) with recovery of 13% protein. Electrophoresis analysis revealed that phytase has molecular weight of 90.5 kDa on non-reducing and 129.6 kDa on reducing SDS-PAGE. The purified phytase exhibited a wider pH and temperature stability. Analysis of the cloned sequence showed that the gene has 1176 bp that encodes for a peptide of 391 amino acids of the core catalytic region. It was also found that phytase from *A. foetidus* has a sequence identity of 99% with the phytase gene of other *Aspergillus* species at nucleotide level and 100% at protein level in *A. niger*, *A. awamori*, *A. oryzae.* In silico analysis of sequence identified the presence of two consecutive and one non-consecutive intra chain disulfide bonds in the phytase. This probably contributed to the differential migration of phytase on reducing and non-reducing SDS-PAGE. There are predicted 11 *O*-glycosylation sites and 8 *N*-glycosylation sites, possibly contributed to an enhanced stability of enzyme produced by this organism. This study opened up a new horizon for exploring the novel properties of phytase for other applications.

## Introduction

Phytate acts as the reservoir of phosphorus in plants and accounts for more than 80% of the total phosphorus (P) in cereals and legumes (Jain and Sapna 2016; Kumar et al. 2017). Despite the usefulness, P remains unavailable to monogastric animals due to the absence of phytases in their gut. Phytates tend to bind to microelements, proteins, carbohydrates, and transfers them into complex insoluble conglomerates (Onyango et al. 2006; Lee et al. 2015). Formation of conglomerates results in decreased bioavailability of P and in turn, unused phytate P is discharged from the gut to environment.

Phytase is a high MW phosphatase that catalyzes the hydrolysis of phytate to myo-inositol and inorganic phosphate (Mullaney et al. 2000). Phytases are found naturally in plants, animals and microorganisms (Wodzinski and Ullah 1996). Fungi, the *Aspergillus* sp. in particular viz., *A. niger*, *A. ficcum* and *A. terreus* are active phytase producers (Vats and Banerjee 2004). Engineered fungal phytases are extensively employed for commercial production. There is ongoing interest (Singh et al. 2011) in augmenting properties of the naturally available phytase. Owing to its ability to release inorganic phosphate, phytase is commonly supplemented in pig and poultry feed. This helps in reduction of additional supplementation of inorganic salts in animal feed, and indirectly reduces environmental pollution due to decrease of phosphorus in fecal excretion. Considering its high environmental and economic importance, it would be imperative to find novel sources of phytase for commercial applications. We previously reported isolation of a novel fungal strain *Aspergillus foetidus* MTCC 11682 from soil. Phytase in crude extract from this strain was able to reduce supplementation of dietary calcium and phosphorus in broiler feed (Manobhavan et al. 2015; Ajith et al. 2018a). Thus, the strain *Aspergillus foetidus* MTCC 11682 was identified to be a potent phytase producer. Here, we report partial purification and molecular characterization of phytases produced from this novel fungal isolate.

## Materials and methods

### Chemicals

Phytic acid sodium salt was purchased from Sigma Chemical, USA. Sephacryl S-200HR was obtained from Amersham Pharmacia Biotech, Sweden. The enzymes and kits for molecular biology experiments were purchased from standard suppliers and used as per manufactures’ instructions. All the general chemicals used for this report were of analytical grade and purchased from either Sisco Research Laboratories or HiMedia Laboratories, Pvt. Ltd, Mumbai, India.

### Fungal strain and culture condition

The spore suspension (1.8 × 10^8^ spores/mL) of *A. foetidus* MTCC 11682 was immobilized on poly urethane foam (PUF) cubes and cultured on an optimized media as per Ajith et al. (2018b). After each cycle of 10 days, the spent culture media were harvested, filtered and filtrates were subjected to assay for phytase activity.

### Assay for phytase activity

Phytase activity was determined colorimetrically by monitoring the release of inorganic phosphorous from phytic acid using Phytex method as described by Kim and Lei (2005).

### Partial purification of phytase

From immobilized fungal fermentation culture, spent media was collected, filtered through Whatman No. 1 filter paper and centrifuged at 10,000 rpm for 10 min at 4 °C. Clear supernatant was adjusted to 90% ammonium sulfate [(NH_4_)_2_SO_4_] saturation for precipitation of proteins. The precipitated proteins was centrifuged at 10,000 rpm for 45 min at 4 °C, solubilized in small volume of 0.01 M sodium acetate buffer (pH 5.0) and desalted by dialysis against the same buffer in refrigerated temperature using 10–14 kDa cut off membrane (HiMedia, Mumbai, India) for 48 h with four buffer changes. The sample was clarified again by centrifugation at 10,000 rpm for 10 min and then filtered through 0.45 μM membrane filter (Uniflow TM, GE Healthcare Life Sciences) and concentrated using Maxidry Lyo^®^ (M/s Heto-Holten, Denmark). After desalting, total protein was quantified by Lowry’s method (Lowry et al. 1951). About 20 mg total protein was further fractionated in Sephacryl S-200 HR column (95 cm × 1.6 cm) using an Automated Biologic Duo-flow system (BioRad, USA) at a flow rate of 1 mL/min in 0.1 M sodium acetate buffer (pH 5.0). The eluted 3 mL per tube fractions were collected for the entire run. Each tube of column eluents was screened at 280 nm for the protein. Phytase activity in each tubes were also screened as per the method described earlier. The column void volume (V_0_) was determined by loading 20 mg dextran blue 2000 (2000 kDa) in 2 mL volume and was used for calibration with standard protein BSA dimer (~ 136 kDa) and monomer (~ 68 kDa).

### Determination of proteins in phytase active column fractions

Elutes in multiple tubes showing phytase activity were pooled, dialyzed in 0.1 M sodium acetate buffer (pH 5.0), concentrated in Maxidry Lyo^®^. Samples were further tested for purity by separating the protein under reducing and non-reducing SDS-PAGE using 10% separating and 4% stacking gel (Laemmli 1970). About 80 μg of column eluted proteins and the standard molecular weight marker was loaded on each well for electrophoresis separation. Following separation, protein bands in gels were stained with Coomassie brilliant blue R-250 and phytase activity was detected by staining procedure (Bae et al. 1999). Gel image was taken in LAS-3000 gel documentation system (M/s Fuji Film, Japan) and the molecular weights of the unknown major and minor protein bands of each lane were determined by comparing the relative front (*R*_*f*_) of the known MW protein markers (Bench Mark unstained protein ladder, Life technologies, USA).

### Effects of temperature, pH, reducing agents and detergents on phytase activity

Phytase activity profile for the temperature optima and thermal stability of the partially purified phytase were determined over a temperature range between 4 and 80 °C for 30 min incubation.

Effect of pH on enzyme activity was determined by pre-incubating the purified enzyme in buffers with pH ranges of 2.5–3.5 (0.2 M glycine), 4.5–5.5 (0.2 M sodium acetate) and 6.5–7.5 (0.2 M Tris HCl) for 6 h followed by phytase assay at 37 °C.

To determine the effect on enzymatic activity, purified phytase was first incubated at 0.05% concentration each of 2-mercaptoethenol (reducing agent), SDS, Triton X-100, Tween-20 and Tween-80 (all detergents) for 15 min at 37 °C and then phytase activity was assayed following the methodology by Kim and Lei (2005).

### Mass spectroscopy analysis of the protein bands

Major protein bands (151.4, 138.5, 121.3, 99.7 and 90.5 kDa) from the gel were excised and washed in 50% acetonitrile solution containing 100 mM ammonium bicarbonate (NH_4_HCO_3_). Samples were reduced using 10 mM dithiothreitol (DTT) in 100 mM NH_4_HCO_3_ solution for 45 min at 56 °C, followed by alkylation using 55 mM iodoacetamide solution in 100 mM NH_4_HCO_3_ for 30 min at room temperature in dark. Finally in-gel digestion was carried out using 20 μL (10 ng/μL) of sequencing grade trypsin in 50 mM NH_4_HCO_3_ overnight at 37 °C. Peptides generated after digestion were extracted in NH_4_HCO_3_ buffer with 5% formic acid. Samples were vacuum-dried and reconstituted in buffer with 5% formic acid. The protein digest spectrum was acquired on M/s Bruker Daltonics Autoflex TOF/TOF mass spectrometer equipped with ion source and FLEX PC instrument. The generated mass (m) to charge (z) ratio peaks were analyzed with the setting of 10% threshold, precursor and fragment mass tolerances of 0.15 Da, cysteine carbamido-methylation as fixed modification and methionine oxidation as variable modification in the Masscot Ion search engine (http://www.matrixscience.com/search_form_select.html).

### Isolation of total RNA and cDNA conversion

About 300 mg mycelium from 18 to 24 h old biomass of *A. foetidus* MTCC 11682 was collected and macerated in liquid nitrogen using a sterile mortar and pestle. To each sample, 2 mL of TriReagent (Sigma Aldrich, USA) was added and then passed through a 20 g needle repeatedly to make it a homogeneous solution. Total RNA from the solution was isolated following Sigma Technical bulletin protocol (https://www.sigmaaldrich.com/content/dam/sigma-aldrich/docs/Sigma/Bulletin/t942bul.pdf). About 2 µg total RNA was reverse transcribed using Reverse Aid H minus first stand cDNA synthesis kit (M/s Fermentas USA).

### Polymerase chain reaction (PCR) amplification, cloning and analysis of sequence

Partial coding region of phyA gene was amplified by PCR using gene specific primers (Forward 5′ GTATCAATGCTTCTCCGAGACTTCG and reverse 5′ CGATCATTAACCAAGACACGGACC) designed from the consensus region of phytase gene coding sequences of four different *Aspergillus* species—*A. niger* (M94550), *A*. *fumigates* (U59804), *A. terreus* (U59805) and *A*. *terreus* (U60412). A typical 20 µL PCR reaction was performed in volume that contained 20 ng total RNA equivalent cDNA, 0.5 µL (5 µM) forward and reverse primers, 10 µL 2XGoTaq^®^ Green master mix. PCR was performed with an initial denaturation at 94 °C for 2 min, followed by 35 cycles each of denaturation at 94 °C for 1 min, annealing at 57 °C for 30 s and extension at 65 °C for 1 min 30 s, and finally an extension at 72 °C for 5 min. A total of 120 µL PCR reaction mix was prepared for the purpose of cloning the fragment. Amplified product was electrophoresed on 1% agarose gel in 1X TAE buffer and visualized with ethidium bromide staining. The desired band was excised and purified using QIAquick gel extraction kit (Qiagen, Germany). About 7 µL of gel purified PCR product was ligated to pJET 1.2 vector as per manufacturer’s protocol, transformed to chemically competent Top10 *E. coli* cells by heat shock treatment and cultured in Luria–Bertani (LB) plate containing Ampicillin (50 μg/mL media). Individual bacterial colonies were picked from the LB plate and were grown overnight in LB broth with Ampicillin (100 μg/mL media). Plasmids were isolated from the grown *E. coli* using QIAprep Spin mini prep kit (Qiagen, Germany). Presence of inserts in plasmids was confirmed by gene specific PCR. Plasmids isolated from three different clones positive for gene specific PCR were further sequenced for both strands by Sanger sequencing reactions with Big Dye Terminator v3.1 Cycle Sequencing method (Applied Biosystems^®^ Inc, USA). Output sequences were derived, confirmed and annotated by homology search using NCBI-BLAST database (https://www.ncbi.nlm.nih.gov/BLAST/). Based on open reading frame, amino acid sequences were deduced and locations of disulphide bonds in the amino acid chain were predicted using online software (http://clavius.bc.edu/~clotelab/DiANNA/). The *N*- and *O*-glycosylation sites were predicted by (http://www.cbs.dtu.dk/services/NetNGlyc) and (http://www.cbs.dtu.dk/services/NetOGlyc) online tools, respectively. The expected amino acid (aa) sequence was deduced. Both nucleic acid and derived aa sequences of nearby species were aligned by CLUSTAL-W program and phylogenetic tree was constructed by neighborhood joining method (http://www.genome.jp/tools/clustalw/). The tree was drawn to scale with branch length in the same units as those of the evolutionary distance of other similar species and synthetic sequences.

## Results

### Purification of phytase

**Fig. 1:**
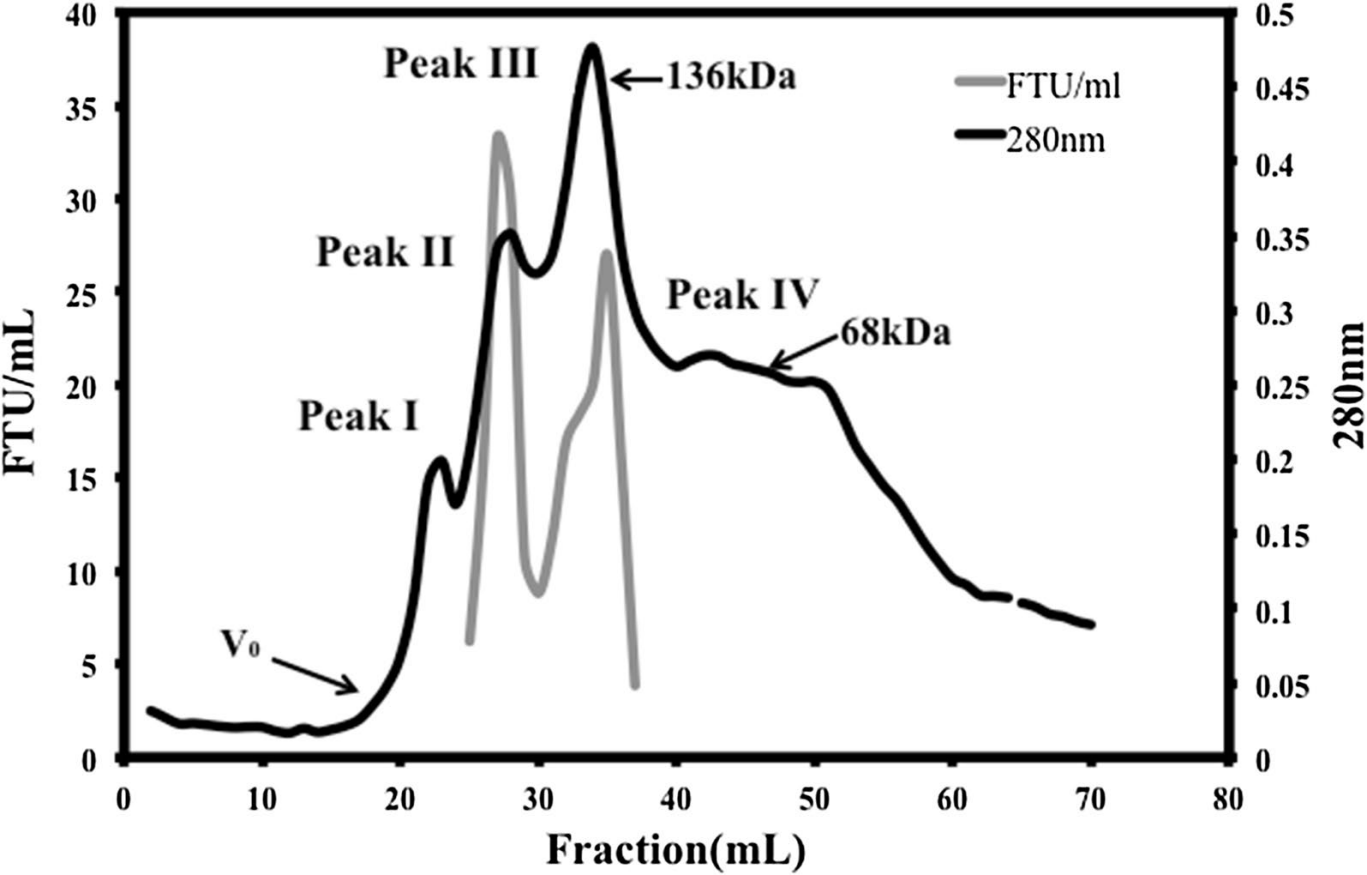
Elution pattern of about 20 mg ammonium sulphate precipitated proteins from fungal spent culture in Sephacryl S-200HR column (95 cm × 1.6 cm) and flow rate of 1 mL per min. The column was equilibrated and eluted in 0.1 M sodium acetate buffer, pH 5.0. All the tubes were monitored for 280 nm absorbance for protein and enzyme activity. Standard protein (BSA) dimer (~ 136 kDa) and monomer (~ 68 kDa) was used to understand the approximate MW range where the phytase active protein eluted

From the crude extract, phytase was gradually purified to a great extent using ammonium sulfate followed by gel filtration chromatography (Table 1). Ammonium sulfate [(NH_4_)_2_SO_4_] precipitate of spent media from *A. foetidus* MTCC 11682 culture showed four protein peaks after fractionation by gel filtration chromatography. Proteins fractionated in peak II and the trailing region of peak III possessed phytase activities (Fig. 1). Thus, combined procedure yielded 23.4-fold purified phytase with recovery of 13% total extracted protein. Partially purified enzyme exhibited improved activity of 295 FTU/mg proteins.

**Table 1:** Total protein and phytase activities in crude culture filtrate, ammonium sulfate precipitate and column elute showing the increase in specific activities with the fold purification of the enzyme produced by *A. foetidus* MTCC 11682 strain

Purification steps	Total protein (mg)	Total activity (FTU)	Specific activity (FTU/mg)	Purification (fold)	Yield (%)
Crude culture filtrate	3600	45400	12.6	1	100
Ammonium sulfate ppt.	245	10200	41.6	3.3	22.5
Sephacryl S-200HR	20	5879	294.5	23.4	12.9

### Effect of temperature, pH, reducing agents and detergents on phytase activity

**Fig. 2:**
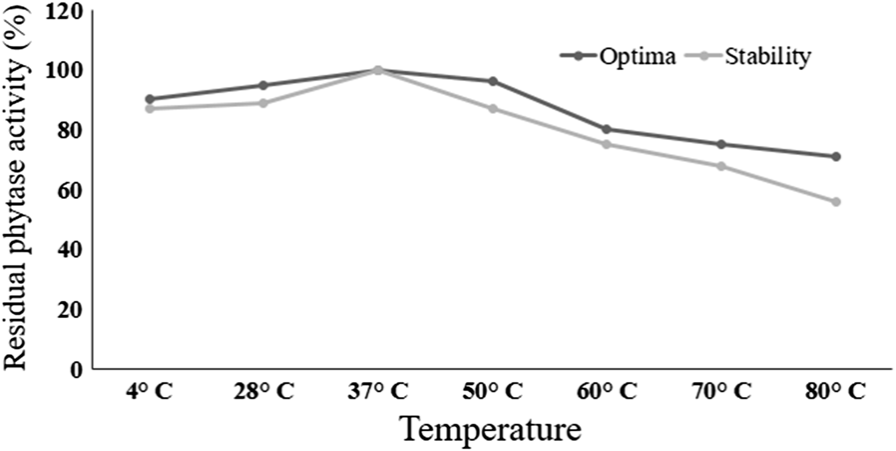
Temperature optima and thermo-stability profiling of purified phytase from *Aspergillus foetidus* MTCC 11682. The 37 °C was found to be the temperature optima and the enzyme was stable for 30 min at temperature ranges between 4 and 60 °C

Phytase isolated from *A. foetidus* MTCC 11682 exhibited activities in the range from 4 to 80 °C with an optimum activity at 37 °C. Activity of enzyme did not change much between 37 and 50 °C. Thermo-stability profiling in terms of maintaining the residual activity indicated that enzyme was stable at 37 °C for 30 min without losing activity whereas at 50 °C the activity retention was 87%. Interestingly, about 56% of residual enzyme activity was retained at 80 °C after 30 min exposure (Fig. 2).

**Fig. 3:**
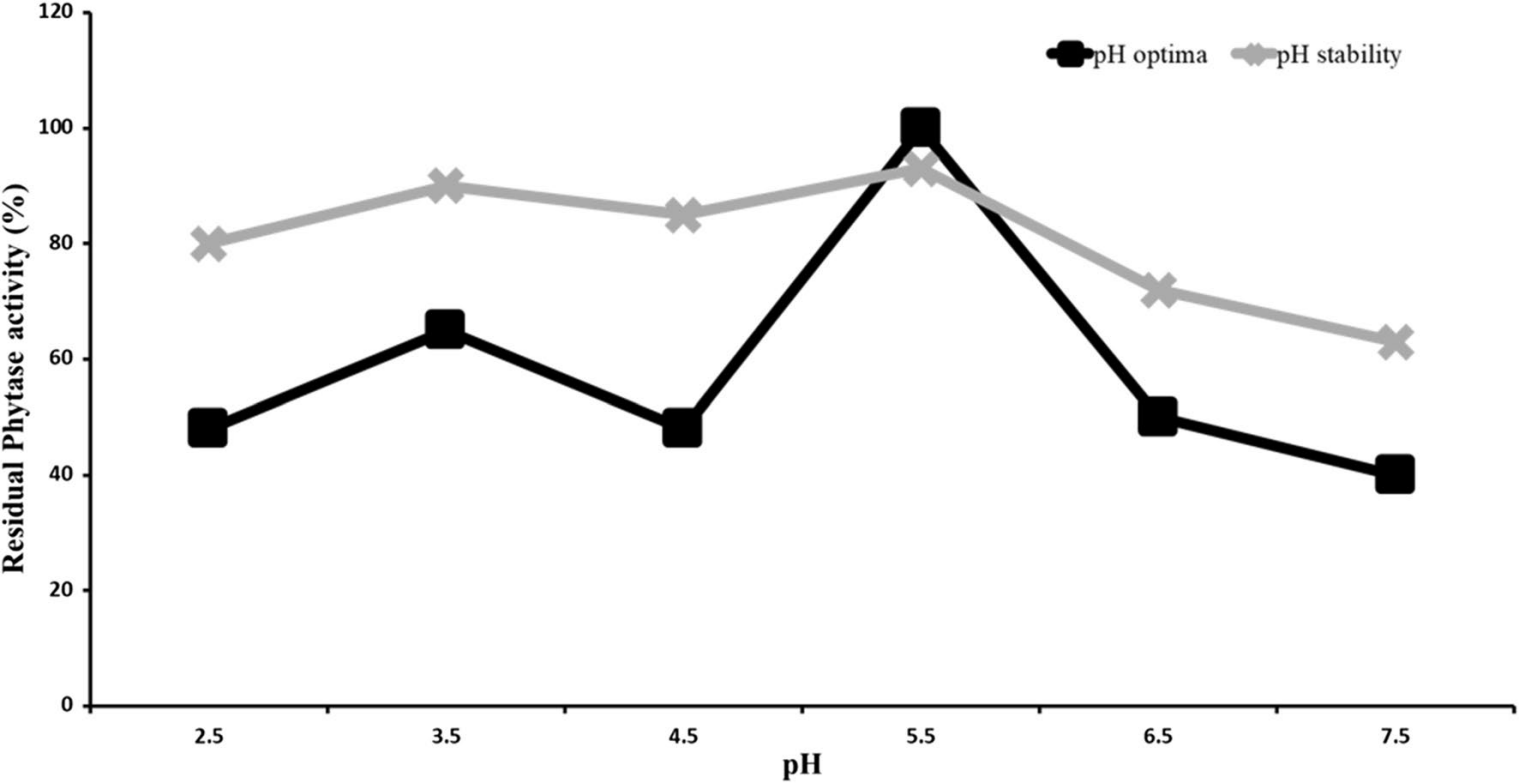
pH profile of purified phytase from *Aspergillus foetidus* MTCC 11682 at incubation temperature of 37 °C. The pH optimum of 5.5 was observed and enzyme was found stable in acidic pH

The enzyme exhibited two peaks of maximum activity at pH 3.5 and 5.5. At these pH values, the enzyme retained 90% of activity even when incubated for 6 h. At pH 5.5, the activity of phytase was 1.5-fold higher compared to its activity at pH 3.5 (Fig. 3). Half of the enzyme activity was lost at pH 2.5, 4.5 and 6.5. A decline in the activity was observed above pH 7.5. The purified enzyme was found to be stable at acidic pH.

**Fig. 4:**
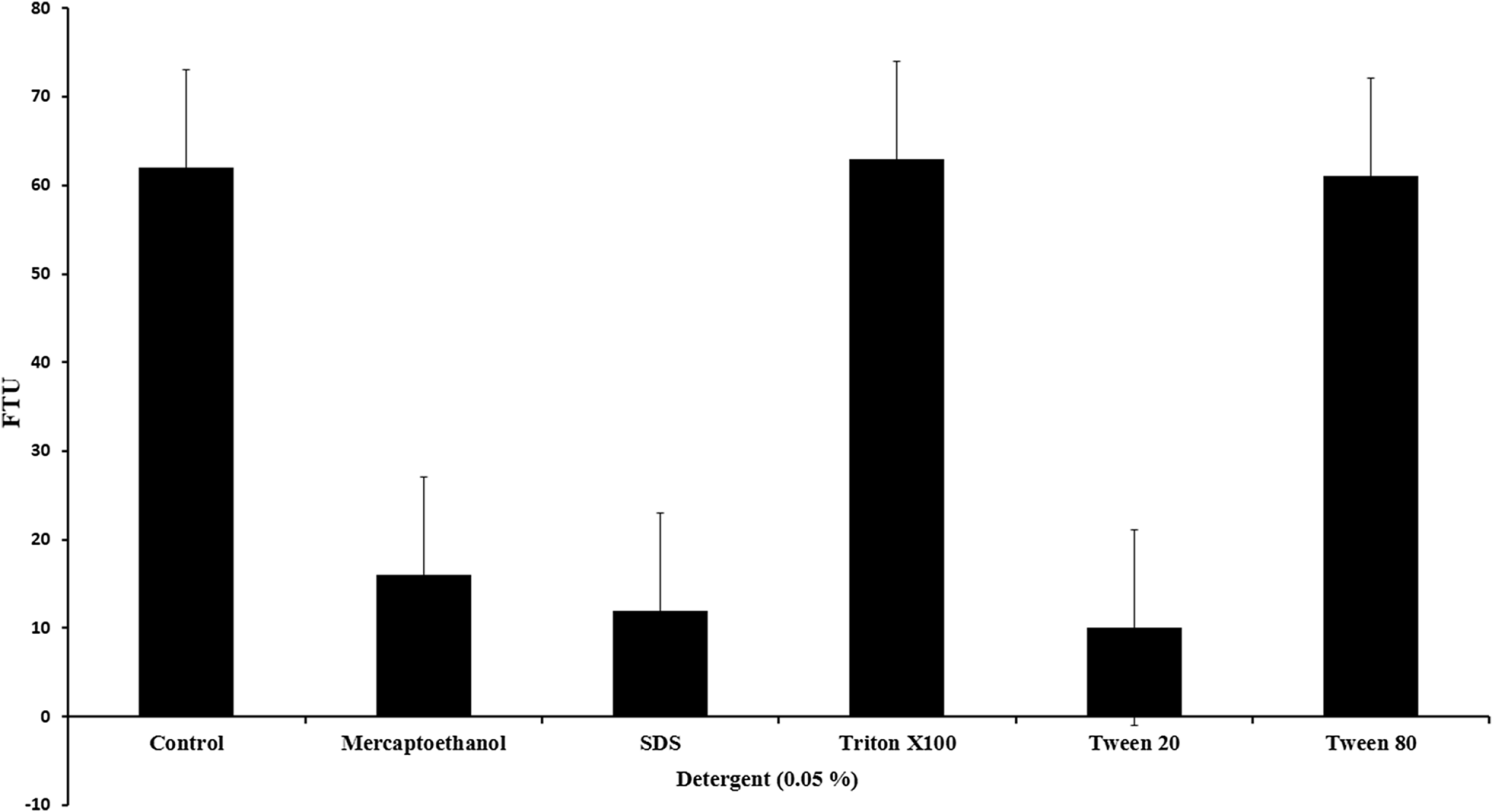
Effect of detergents on purified phytase from *Aspergillus foetidus* MTCC 11682. No detergents exhibited an inducing effect on the phytase activity however, mercaptoethanol, SDS and Tween 20 showed an inhibitory effect

The detergents Triton X-100 and Tween-80 at 0.05% inclusion level showed no effect on phytase activity as compared to that of control. However the reducing agent, 2-mercaptoethanol, and denaturing agents’ viz., SDS and Tween-20 at similar inclusion levels showed inhibitory effects on the phytase activity (Fig. 4).

### Molecular mass of the phytase enriched fractions

**Fig. 5:**
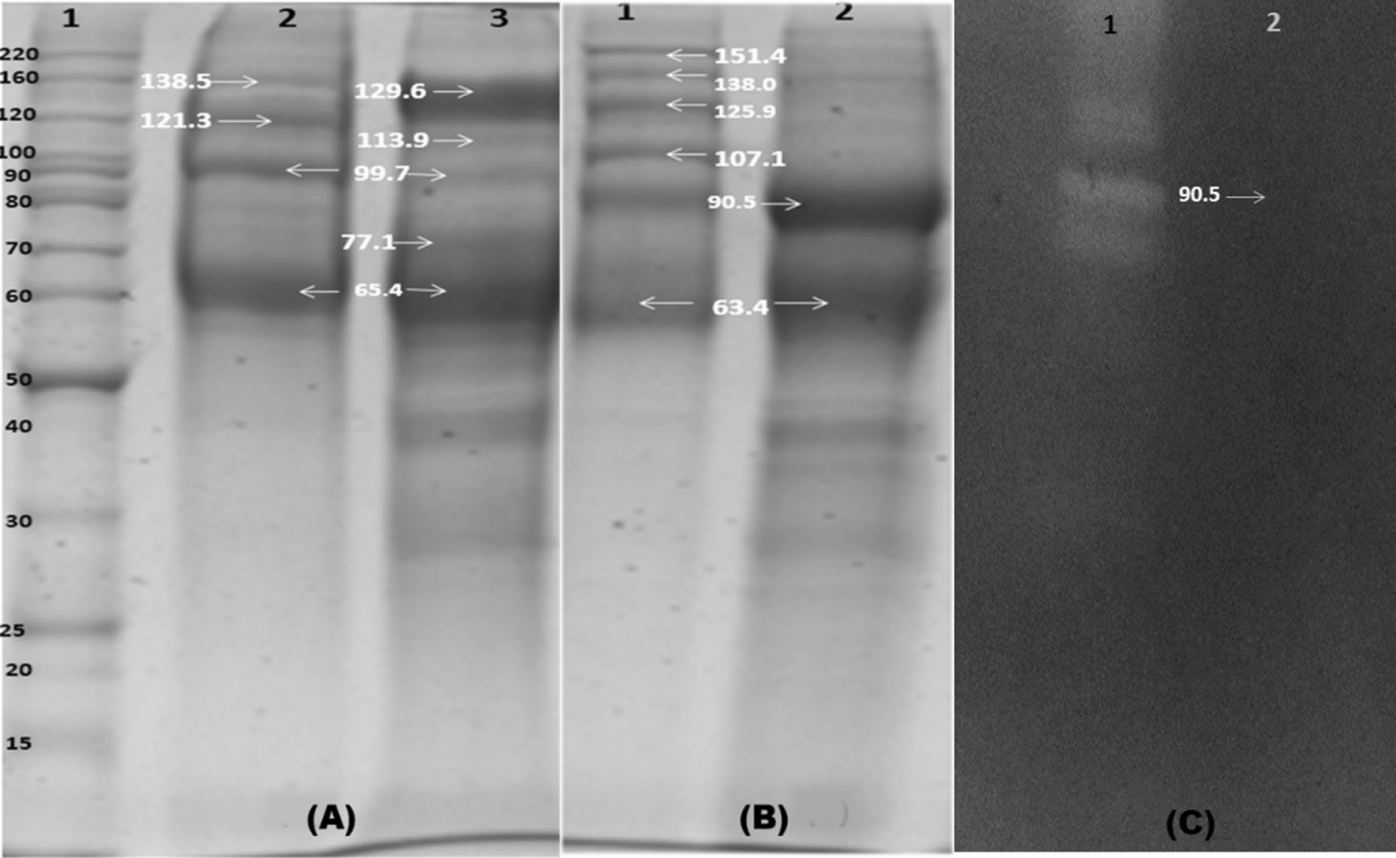
Coomassie Blue stained 10% SDS-PAGE gel image showing the protein profiles under **A** reducing **B** non-reducing conditions **c** zymogram for activity bands of partially purified protein eluted by Sephacryl S-200HR column. In panel (**A**), Lane 1 is the standard molecular weight (MW) marker protein and lane 2 and 3 represents the peak II and III proteins subjected to 2 Mercaptoethanol treatment. Shifting of the high intense band in lane 3 indicated the presence of disulfide bond in the protein. In **B**, lane 1 and 2 represents peak II and III proteins without 2 ME treatment. **C** represents the zymogram showing the phytase activity. Lane 1 clearance zone denoted by positive standard phytase (*E. coli* produced recombinant phytase of *A niger*) and lane 2, though the clearance zone could not be captured, the phytase activity band was observed at the arrow head

The proteins in peak II exhibited higher molecular mass (151.4, 138.0, 125.9, 107.1, 90.5 and 63.4 kDa) as compared to the lower molecular mass (major 90.49, 63.4 kDa and many other minor lower MW bands) of proteins in trailing peak III (Fig. 5B). A 90.5 kDa common band was detected in elutes from both peaks. When treated with 2-mercaptoethanol (2-ME), proteins of size 138.5, 121.3, 99.7 and 65.4 kDa bands appeared in peak II elute whereas, elute of peak III showed a major 129.6 kDa band. Thus, proteins from both elutes, we detected a band of 90.5 kDa in non-reducing condition and the same band migrated as 129.6 kDa after 2-ME reduction (Fig. 5A, B). In addition, zymogram analysis revealed that this 90.5 kDa protein (non-reduced form) from peak III elute only possessed clearance activity of phytase (Fig. 5C).

### Sequence characteristics of the cloned phytase gene

**Fig. 6:**
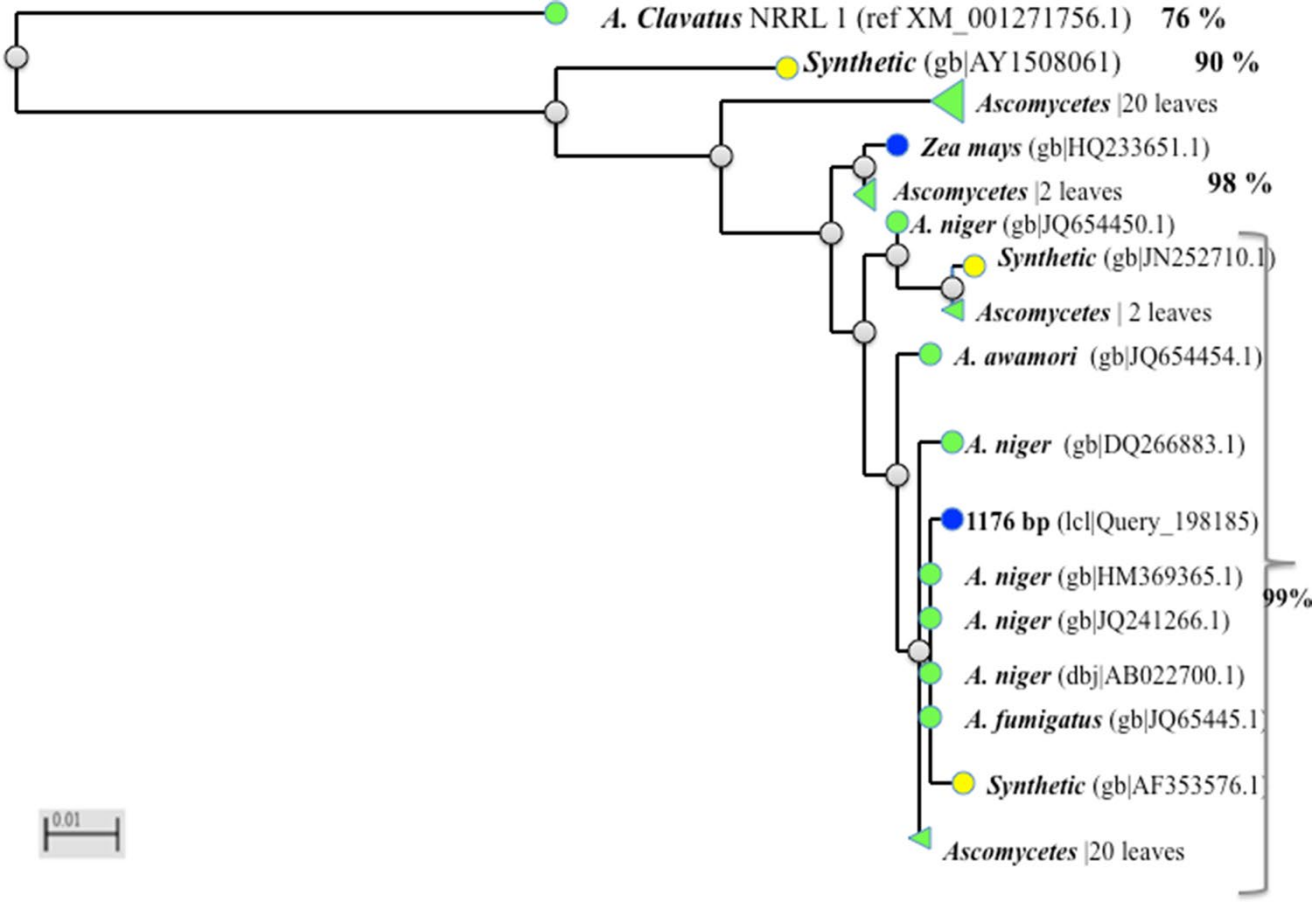
Phylogenetic relationship of 1176 bp cloned partial phytase gene complementary DNA sequences of *Aspergillus foetidus* MTCC 11682 with different other close by species and synthetic sequences. Relationship tree was constructed by neighbourhood joining method using NCBI Blast tree view software with default setting. The percentage similarity of sequences is given to the right

PCR products were cloned in plasmids and three different clones were sequenced. It was found that each of those clones had an identical 1176 bp long PCR product. The nucleotide sequence encoded for 391 amino acids belonging to the core catalytic regions of the functional phytase. The nucleotide sequence was submitted to the NCBI GenBank database (accession number KY307787). The NCBI-BLAST analysis (Table 2) revealed the sequences had 99% nucleotide identity with the PhyA gene of *Aspergillus* genus. However, at protein level it had 100% homology with phytase from key producing organisms viz., *A. niger*, *A. awamori*, *A. oryzae* and 99% sequence identity with phytase sequences from *A. ficcum*, *A. usamii* and *A. fumigatus* (Fig. 6).

**Table 2:**
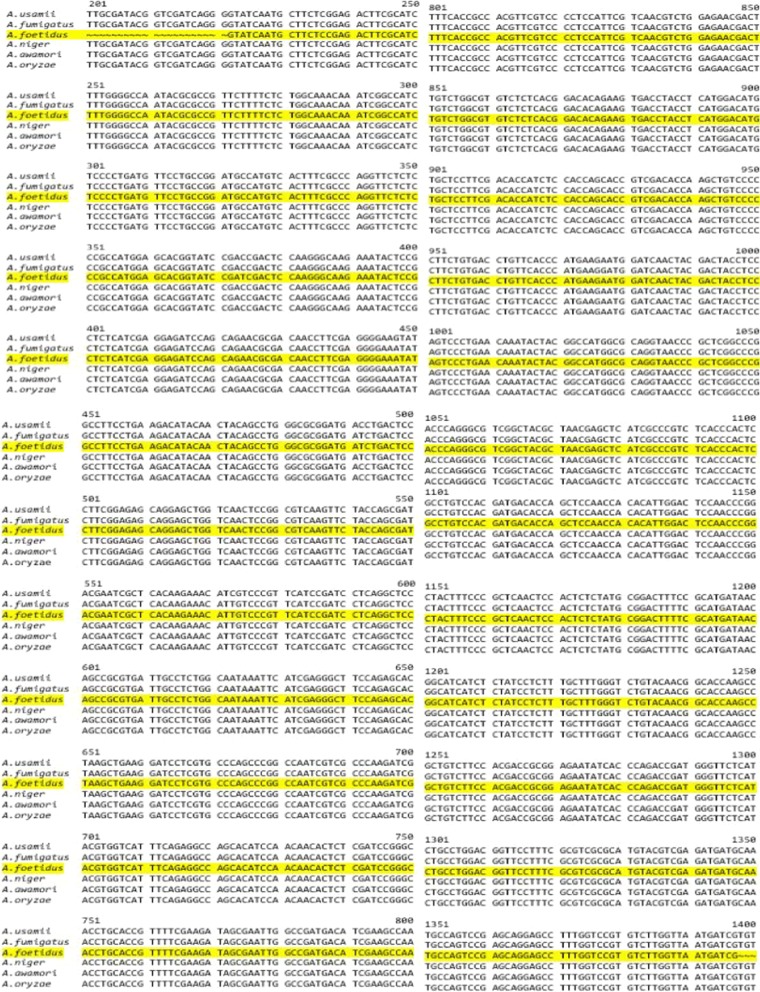
Nucleotide sequence of *A. foetidus* MTCC 11682 (Acc. No. KY307787) was compared with other *Aspergillus* sps. like *A. usamii* (DQ198163), *A. fumigatus* (JQ654451), *A. niger* (HM369365), *A. awamori* (DQ192035) and *A. oryzae* (AY603416) using Clustal omega (NA_MULTIPLE_ALIGNMENT 1.0, squid. MSF) showing 100% similarity

In Pfam the sequence was found to be similar to the histidine acid phosphatase (HAP) protein family. The catalytic domain of HAP and phytases (myo-inositol hexakisphosphate phosphohydrolase) shared a common feature.

Mass spectrometry analysis of 151.4, 138.5, 121.3, 99.7 and 90.5 kDa bands by MALDI TOF/TOF could not resolve the identity of phytase protein in any of the band.

Analysis of the cloned sequence revealed presence of probable 8 *N*-glycosylation and 11 *O*-glycosylation sites in the cloned functional region sequences (Table 3).

**Table 3:**
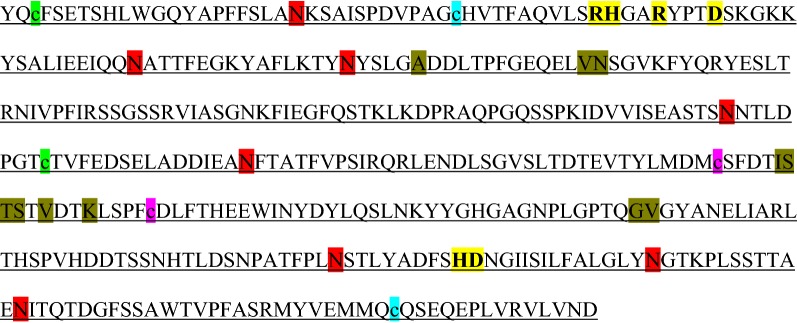
Derived 391 partial amino acid sequences showing 3 predicted intra chain disulphide bond locations denoted in small letter case (c) and in different highlight colors, 8-*N* glycosylation sites with red high lights and 11-*O* glycosylation sites and location of catalytic domain of the enzyme with bold and yellow high lights

## Discussions

Purification of phytase enzymes from novel sources has paramount importance for multiple downstream applications. This is the first report on partial purification of phytaseA from a novel fungal strain, *Aspergillus foetidus* MTCC 11682. We demonstrate that precipitation of crude extract with ammonium sulfate followed by gel chromatography improves purification of enzyme by several folds and the process does not affect enzymatic activity. We further provide data on chemical properties of this fungal enzyme.

In our experiment with extracts of *A. foetidus*, we noted dual protein peaks with phytase activity, presumably presence of two different proteins with different molecular weights, similar to previous report with *A. niger* NCIM563 (Soni et al. 2010). Peak II proteins had molecular weights between 136 kDa (BSA dimer) and 250 (upper limit of separation range) whereas, proteins from peak III elute had molecular weights between 68 (BSA monomer) and 136 kDa (BSA dimer), indicating the phytase activity emanates from monomer (peak III) and dimer (peak II). It is worthy to note that dimer formation indeed occurs in phytase of other fungal strain *A. niger* UFV (Monteiro et al. 2015). Given the fact that dimer of phytase from *A. niger* UFV, a thermotolerant strain has molecular weight of 161 kDa (Monteiro et al. 2015), it is likely that dimer from *A. foetidus* has a similar molecular weight of 151.4 kDa (Fig. 5B).

Depending upon the presence of intra chain disulfide bonds, same protein, owing to their different secondary structures, migrates differently in non-reducing SDS-PAGE gel. The present study reports on presence of intra chain disulfide bonds (Table 3), testified by appearance of multiple higher molecular weight proteins (non-reducing SDS-PAGE gel, Fig. 5B), and disappearance of most of those bands to a minimal number (reducing SDS-PAGE gel, Fig. 5A).

When compared band profiles of proteins in peak III (which showed most phytase activity), we noted a prominent band of size 90.5 kDa in the non-reducing SDS-PAGE gel however, this band disappeared in the reducing SDS-PAGE gel. Instead a band with MW 129.6 kDa was detected after 2-ME treatment. The 90.5 kDa also displays phytase activity in zymography (Fig. 5). Taken together, the data suggest that phytase in *A. foetidus* fungal strain has a MW of 90.5 kDa (native conformation) and upon reduction of disulphide bond by 2-ME, it gives rise to a 129.6 kDa (linear conformation) band. This data also indicated that *A. foetidus* phytase may not be far different from that of reported range of MW (84–87 kDa) in other strains of fungus *A. Niger* (Casey and Walsh 2003; Greiner et al. 2009) or any other phytase previously purified (MW ranges 38–200 kDa, Ullah 1988; Dvorakova et al. 1997; Skowronski 1978). Appearance of some other insignificant bands (low intensity 113.9, 99.7, 77.1 kDa bands; and high intensity 63.4 and 65.4 kDa bands) seem to be contaminants since the molecular weights remain unchanged after reduction. Thus, comparing the identical *R*_*f*_ values in the non-reducing SDS-PAGE and zymogram, we conclude that phytase protein of this organism appeared at 90.5 kDa.

Purification procedure applied in the current report recorded a 23-fold increase in the specific activity of phytase, like the other published work (Casey and Walsh 2003; Neira-Vielma et al. 2018; Monteiro et al. 2015). Interference by contaminant proteins was undetectable and partially purified enzyme was sufficient for studying stability parameters. Supplementation of broiler feed with desalted ammonium sulfate precipitate of crude culture fungal extract (containing partially purified phytase) significantly improved P utilization in animals, reducing P excretion to the environment (Ajith et al. 2018a). This data indicate that for using the same product in commercial broiler feed additional gel filtration column elution step may not be required, unless a reasonably high specific enzyme activity needed for other applications.

The enzyme remained active at temperature range between 37 and 50 °C and unlike other reports (Vats and Banerjee 2004; Greiner et al. 2009). Phytase purified from *A. foetidus* strain had higher thermo-stability compared to enzymes isolated from other fungi (Casey and Walsh 2003; Greiner et al. 2009), though inferior to phytase isolated from *A. niger* UFV-1 (Monteiro et al. 2015). The thermo-stability of this phytase could probably be due to the operational stability acquired from immobilization of fungus (Klein and Wagner 1983; Westman et al. 2012). This property could be useful for using the enzyme as supplement to animal feed.

The isolated phytase displayed dual pH optima similar to enzymes from different strains of *A. niger* (Wodzinski and Ullah 1996; Sariyska et al. 2005; Greiner et al. 2009). The enzyme activity declined from pH 7.5 onwards, like other reports (Oh et al. 2004). Based on pH optima the phytase described here belongs to PhyA group (2.5 and 5.5 pH) (Soni and Khire 2007; Soni et al. 2010). Based on conserved motifs, it is grouped into histidine acid phytase (HAP) (Mullaney and Ullah 2003). The pH profile of enzyme activity, conserved motifs from in silico analysis leads to place this enzyme under HAP PhyA group. This property of activity optima in acidic pH helps in extending its application as feed additive for the release of phosphate from feed phytate in the digestive tract of salivary gland (5.0), stomach (2.0–4.0) and small intestine (4.0–6.0) (Lindberg and Ogel 2001).

In our study we observed enzyme remains stable even when exposed to different strong reagents such as TritonX100 and Tween 80. This enhanced stability of this enzyme could be attributed to the glycosylation, similar to those observed in recombinant phytase (Guo et al. 2007; Gebert et al. 2015).

We predicted three disulfide bridges in the amino acid backbone of the cloned functional phytase and this explains the differential migration of phytase proteins in non-reducing and reducing gel electrophoresis. A LC–MS/MS based mass spectrometry and de novo sequencing approach instead of simple MALDI–TOF/TOF method would be required to reveal the actual identity of the protein with phytase activity. (Wyss et al. 1999).

Minor differences in molecular weights of phytase isolated from this fungal species could be due to species difference in the non-conserved regions sequences as the cloned conserved region was similar (Table 2). Species specific differences in the N-terminal region of sequence for this gene are reported in other *Aspergillus* sp. (Wyss et al. 1999). The failure in obtaining the complete N-terminal sequence by placing the left primer on sequence of other species phytase gene and the right primer in the conserved region probably indicated sequence differences with the reported species (data not shown).

In conclusion, we demonstrated here that *Aspergillus foetidus* MTCC 11682 produces uniquely stable acidic phytase enzyme, PhyA. Adaptation of immobilization technique of the organism (external factor) for production of fungal phytase and glycosylation of the molecule (internal factor) are key factors likely to contribute to enhanced stability of this enzyme. Production of recombinant enzyme in other (heterologous) hosts should be taken up for enhancing desirable features of this enzyme through regulating glycosylation event.

## Abbreviations

MTCC: Microbial Type Culture Collection
P: phosphorus
PUF: poly urethane foam
BSA: bovine serum albumin
FTU: phytase units
ME: mercaptoethanol
HAP: histidine acid phosphatase
